# Validation of a face image assessment technology to study the dynamics of human functional states in the EEG resting-state paradigm

**DOI:** 10.18699/VJGB-22-92

**Published:** 2022-12

**Authors:** A.N. Savostyanov, E.G. Vergunov, A.E. Saprygin, D.A. Lebedkin

**Affiliations:** Institute of Cytology and Genetics of the Siberian Branch of the Russian Academy of Sciences, Novosibirsk, Russia Scientific Research Institute of Neurosciences and Medicine, Novosibirsk, Russia Institute for the Humanities of Novosibirsk State University, Novosibirsk, Russia; Scientific Research Institute of Neurosciences and Medicine, Novosibirsk, Russia; Institute of Cytology and Genetics of the Siberian Branch of the Russian Academy of Sciences, Novosibirsk, Russia Scientific Research Institute of Neurosciences and Medicine, Novosibirsk, Russia; Scientific Research Institute of Neurosciences and Medicine, Novosibirsk, Russia Institute for the Humanities of Novosibirsk State University, Novosibirsk, Russia

**Keywords:** neurocognitive studies, own and other face, EEG correlates, covariates, implicit cognitive processes, self-perception, нейрокогнитивные исследования, свое и чужое лицо, ЭЭГ-корреляты, ковариаты, имплицитные когнитивные процессы, самовосприятие

## Abstract

The article presents the results of a study aimed at finding covariates to account for the activity of implicit cognitive processes in conditions of functional rest of the subjects and during them being presented their own or someone else’s face in a joint analysis of EEG experiment data. The proposed approach is based on the analysis of the dynamics of the facial muscles of the subject recorded on video. The pilot study involved 18 healthy volunteers. In the experiment, the subjects were sitting in front of a computer screen and performed the following task: sequentially closed their eyes (three trials of 2 minutes each) and opened them (three trials of the same duration between periods of closed eyes) when the screen was either empty or when it was showing a video recording of their own face or the face of an unfamiliar person of the same gender as the participant. EEG, ECG and a video of the face were recorded for all subjects. In the work a separate subtask of the study was also addressed: validating a technique for assessing the dynamics of the subjects’ facial muscle activity using the recorded videos of the “eyes open” trials to obtain covariates that can be included in subsequent processing along with EEG correlates in neurocognitive experiments with a paradigm that does not involve the performance of active cognitive tasks (“resting-state conditions”). It was shown that the subject’s gender, stimulus type (screen empty or showing own/other face), trial number are accompanied by differences in facial activity and can be used as study-specific covariates. It was concluded that the analysis of the dynamics of facial activity based on video recording of “eyes open” trials can be used as an additional method in neurocognitive research to study implicit cognitive processes associated with the perception of oneself and other, in the functional rest paradigm.

## Abstract (ru)

В статье представлены результаты исследования, направленного на поиск ковариат для учета деятельности имплицитных когнитивных процессов в условиях функционального покоя испытуемых и при демонстрации им собственного или чужого лица в совместном анализе данных ЭЭГ-эксперимента. Предлагаемый подход основан на анализе динамики мышц лица испытуемого по видео. В пилотном исследовании приняли участие 18 здоровых добровольцев. В эксперименте испытуемые, сидя перед экраном, последовательно закрывали глаза (три пробы по 2 минуты) и открывали их (также три пробы между периодами закрытых глаз) либо перед пустым экраном, либо перед экраном с демонстрацией видеозаписи их собственного лица или лица незнакомого им человека такого же пола, что и участник. У всех испытуемых регистрировали ЭЭГ, ЭКГ и вели запись видео лица. В работе решали отдельную подзадачу эксперимента: апробацию методики оценки динамики активности мышц лица испытуемых по их видео с открытыми глазами для получения ковариат, которые можно включать в последующую обработку совместно с ЭЭГ-коррелятами в нейрокогнитивных экспериментах с пара- дигмой, не предполагающей выполнение активных когнитивных заданий (resting-state conditions). Показано, что пол испытуемого, статус экрана (пустой, собственное/чужое лицо), номер пробы связаны с различиями в мимической активности лица и могут выступать искомыми ковариатами. Сделан вывод, что анализ динамики мимической активности по видео с открытыми глазами может быть дополнительным методом в нейрокогни- тивных исследованиях для изучения имплицитных когнитивных процессов, связанных с восприятием изобра- жения себя и другого, в парадигме функционального покоя.

## Introduction

Technologies of neurocognitive studies are most often based on the use of various approaches to recording the brain activity of experiment participants using techniques such as EEG or fMRI (Bringas-Vega et al., 2022). In the last two decades (Biswal, 2012; Snyder, Raichle, 2012) the researchers’ interest has been focused on the functional states of the brain observed in the absence of exogenous cognitive or emotional load, that is, in the experimental paradigm of “resting-state conditions”.

In a series of studies, it was shown that the functional states of the brain at rest reflect the individual characteristics of the subjects, including their gender (Volf et al., 2015), age (Privodnova et al., 2020; Engemann et al., 2022), genetic features (Proshina et al., 2018), sociocultural affiliation (Knyazev et al., 2012), climatic and geographical living conditions (Milakhina et al., 2020), psychological personality traits (Kabbara et al., 2020) and predisposition to affective disorders (Greicius et al., 2007). However, the problem of using neuroimaging techniques consists in the high variability of resting-state brain activity characteristics in healthy subjects (Li et al., 2022). A comparative study by M. Li and colleagues, performed on a sample of more than 1500 participants in nine countries, showed that the resting-state EEG characteristics of a healthy person vary greatly depending both on the characteristics of the subjects and on the conditions in which imaging sessions take place which are not specified in the experimental paradigm (Li et al., 2022). At the same time, formally the same EEG recording conditions (closed eyes without external mental load) can give different results depending on the part of the world and the period of the year the EEG was recorded in.

One of the factors that significantly changes the functional states of the brain at rest is the presence or absence of the person’s thoughts about themselves during the period of registration of their brain activity. In the work (Knyazev et al., 2012) it was shown that thinking about oneself induces increased activity of the default mode brain network. At the same time, the functional organization of the default system under these conditions demonstrated significant intercultural differences when comparing subjects from Novosibirsk and Taiwan.

In the case of fMRI, an additional factor is the person’s response to the very situation of placing them in the scanner. The fMRI recording is done while the person is lying in a confined tube with sound-induced noise and limited mobility, and sometimes contrast agent injection is required. Obviously, some people react to such conditions as a stressor, while other people perceive these conditions differently, which causes a wide spread in the assessments’ results of the subjects’ functional state. Hence, the task arises: on the basis of additional methods, to find such correlates (or covariates) that, during subsequent analysis, together with the results of EEG or fMRI examinations, will allow to more precisely account for the psychophysiological state of the subject.

In the case of an experimental paradigm using stimuli to induce the desired state of the participants, the assessment of such a state is done by analyzing behavioral indicators (for example, the accuracy/speed of response to external stimuli), but in the case of the resting-state studies, this is not possible.

Another method consists in the usage of psychological questionnaires that the participant is asked to complete before or after the experiment session. Questionnaire indicators are used as variables to assess the subjective states of a person under experimental conditions or their personality traits. However, this method is limited by the sincerity of the test subject and their ability for adequate self-assessment, which can be pronounced in the case of neuropsychiatric diseases.

In our pilot study, we propose an approach using covariates that can be obtained from the dynamics of facial muscle activity recorded on video and are associated with the psychophysiological state of the participants in the EEG experiment. The analysis of facial muscle activity in psychophysiology has been tested (Nikolaeva, Vergunov, 2021), but has not been used for joint analysis with EEG data.

We test the hypothesis that the subjects’ facial activity dynamics and the duration of the eyes screen fixation in resting-state activity sessions with the absence of explicit experimental tasks differ depending on the factors such as the subject’s gender, the demonstration of a blank screen or a screen with a video of their own face or a face of another person of the same gender, the order of experiment stages (“blank screen”, “own face”, “other face”).

The participants were subjected to complex psychological testing to assess their personality traits with co-registration of EEG, ECG and video recording of facial activity. However, within the framework of this study, we will not present the results of EEG, ECG, and psychometry, leaving them for future joint analysis with the identified covariates at subsequent stages of the experiment.

## Materials and methods

Sample description. The experiments involved 18 volunteers (8 men and 10 women, mean age 19.5 ± 1.3 years), all students of Novosibirsk State University. Before the survey, all participants signed an informed consent form. In addition, all subjects completed a questionnaire for the presence of psychiatric or neurological diseases, a questionnaire for wellbeing before the examination, and for the use of alcohol or psychoactive substances. The exclusion criteria were: – certain established medical diagnoses; – use of drugs or psychotropic medications; – a state of alcoholic intoxication or severe psychological stress; – violation of the instructions during the experiment session (covering part of the face with a hand, sudden movements, changing the posture so that part of the face goes out of the camera frame, etc.).

Experiment session procedure. During the experiment session, the subjects sat in a chair in a soundproof chamber with subdued lighting. An EEG helmet was placed on the participant’s head, and electrodes were attached to the left arm and both legs for ECG recording. The subjects were informed that in the process of recording EEG and ECG, a video recording of their face was being made. The protocol of the experiment was approved by the ethical committee of the Scientific Research Institute of Neurosciences and Medicine in accordance with the ethical standards of the Declaration of Helsinki for biomedical research.

Participants were instructed to minimize movement of their arms, legs, and head. During the EEG recording session they had to, on command given by the computer, open or close their eyes. Participants were not specifically required to focus their eyes on the screen, but they were not prohibited from doing so. Each participant was tested in three different conditions: a) background recording with alternating eye closing/opening (3 trials of each type for 2 minutes), in which there were no images on the computer screen; b) recording with opening and closing of the eyes, in which a video recording of the participant’s own face, made earlier during condition (a), was shown on the screen (3 trials of each type for 2 minutes); c) recording with opening and closing of the eyes, in which the participant was shown a video recording of the face of a person he did not know, but of the same gender as the participant (3 trials of each type for 2 minutes).

All participants were examined in all three conditions. The first condition has always been the (a) condition, i. e., recording without additional external stimulation, for half of the participants the second condition was (b) (own face), and the third was (c) (another face), and for the other half of the participants, on the contrary, the second was the condition (c), and the third was (b).

In between these recordings, participants performed active experimental tasks – solving linguistic tests for finding syntactic errors in sentences between the first and second examinations (approximately 25 minutes) and performing motor tests in the stop-signal paradigm (approximately 12 minutes) between the second and third examinations.

Before the first experimental condition, all participants filled out the Russian version of the C. Spielberger questionnaire to assess the level of situational anxiety (Khanin, 1976). After completion of the first condition, the C. Spielberger questionnaire was filled out again to assess whether participation in the survey affects the level of situational anxiety. In addition, after completing each of the experimental conditions, the participants filled out a G.G. Knyazev questionnaire on well-being during the EEG recording (Knyazev et al., 2012). Thus, each participant filled out the C. Spielberger questionnaire twice (before and after the first experimental condition), and G.G. Knyazev three times (after each experimental condition)

Our proposed study design allows to control the factors that may accompany implicit cognitive processes taking place during presentation of faces (one’s own and other’s) or a blank screen: – features of the motor units activity for the face muscles (AU) according to Facial Action Coding System (FACS); – features of time distribution in the test in relation to the subject’s gaze fixation on the screen; – features of perception for subjects of different genders; – features of perception for the first and subsequent conditions; – individual specificity of implicit cognitive processes associated with the personality traits of the subjects, such as the level of anxiety

Note that the analysis of the last factor (individual specificity) is not included in the objectives of this study. Later, a joint analysis of the results of psychological questionnaires with the results of clustering statistics for this factor will be used for psychophysiological profiling of the subjects.

**Fig. 1.**
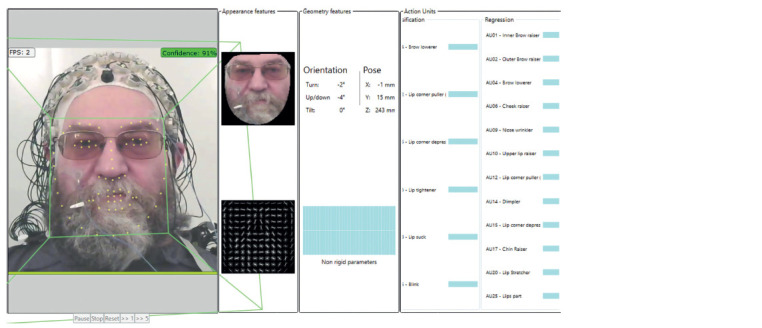
A screenshot of the program for the processing of facial expressions of the participant’s face from the video with added complex analysis-wise elements (dark glasses, beard, mustache, cap with electrodes for EEG recording). FPS – processing speed, number of frames per second; confidence – the level of reliability (green indicates an acceptable level); appearance features – features of facial expressions recognized by the program after bringing the face to a vertical position; geometry features – 3D geometry of the position and orientation of the face; action units is the activity of motor units for facial muscles (AU according to FACS); classification – AU values obtained by the classification method (not used in this study); regression – AU values obtained by the regression method (see the Table); orientation – angular 3D indicators of face orientation; turn – face rotation (left+, right–); up/down – tilt of the face (up+, down–); tilt – tilt of the face to the shoulder (left+, right–); pose – linear indicators of the position of the face; X, Y, Z – coordinates of the center of the face (in mm); non rigid parameters – soft parameters; pause, stop, reset, >>1, >>5 – frame/video player control buttons.

Method for assessing the expression of facial muscles. Specialized software tools, including those in open access, are being widely developed to assess the expression of facial muscles of the subject from video. The OpenFace framework was used in this study – an open access solution that allows to highlight a person’s face from an image, from a sequence of images or from a video stream (Saprygin et al., 2022). A video stream was recorded on a regular computer video camera (webcam) when the subjects performed tasks, then, based on the regression model, facial motor units (AU) were identified using the FACS system (facial action coding system) and the dynamics of their activity during tests with open eyes were analyzed (Fig. 1). Empirically, it was found that the dynamics of AU during a period of about two minutes of immobile sitting of the subject is best characterized not by the average value or standard deviation (a large number of small random changes create “noise”), but by the range of values. Therefore, exactly the range of expression values for each AU was included into the analysis.

The OpenFace framework is based on the CLM (constrained local model) approach. The pilot software developed by the authors based on OpenFace_GUI allows real-time visualization of a set of features provided by 3D models of the OpenFace framework (coordinates of key points of the face, position and angles of the head in space, direction of gaze). The OpenFace framework consists of three main parts: 1) C++ code in which the main analytical flow is implemented; 2) files of pre-trained models for face detection, detection and tracking of key points of the face, calculation of motor units; 3) Matlab code to create your own model files.

Model files are created using a wide variety of training datasets. The OpenFace framework code is open source and available under the GNU license: https://github.com/TadasBaltrusaitis/ OpenFace.

Mathematical foundations of the model. PLS-analysis is a method of obtaining projections on latent structures, the original name of which is “partial least squares method”. An effective tool for PLS analysis is 2B-PLS models (2B- PLS, two-block PLS) (Rohlf, Corti, 2000). 2B-PLS models being applied to the study of implicit cognitive processes reveal deep independent (orthogonal) “latent structures” (psychophysiological mechanisms) simultaneously for two different blocks (matrices B1 and B2) of multidimensional indicators (Kovaleva et al., 2019).

When constructing 2B-PLS models, the data series are centered, both blocks are scaled and rotated to obtain the maximum covariance between the score matrices (B1- and B2-score), which are projections of the matrices B1 and B2 onto the desired latent structures. This is the main difference between 2B-PLS and PCA (principal component analysis, the method of principal components), which allows you to build models only of a “single-component” type. For example, one block can contain feature variables (consisting only of “0” and “1”, the variance is minimal), and the other-rows of instrumental data (in which the variance is much larger than that of the features).

The latent structures obtained in the 2B-PLS model are described using orthogonal load matrices (B1- and B2-loadings). Rows in matrices B1 and B2 are objects’ data, columns are the indicators. Thus, indicators act as initial coordinate axes (including those correlated with each other), and can be considered as “explicit structures”, each of which determines a certain (usually small) amount of total variance. The purpose of the 2B-PLS model is to find a system of pairs of axes for both blocks at once, which express the maximum covariance pattern (Polunin et al., 2019). At the same time, the load matrices are the transition matrices from the original “explicit structures” to the newfound “latent structures”.

As a result of applying a 2B-PLS model, we get the number of latent structures (new coordinate axes), which is equal to the minimum number of variables from the two blocks of initial data. Note that the ratios for raw data structures in blocks remain the same after any number (and order) of application of operations such as centering, scaling and rotation, which are applied in PLS models or PCA models. Thus, the structure of the raw data is completely preserved, while the tools of the least squares method (ordinary least squares, OLS) in some cases can lead to alteration of the original structure.

As a result of building a 2B-PLS PLS model, all information from the initial data series (the number of which can be hundreds or more) is collected into the first few independent latent structures. 2B-PLS model allows for a situation where the number of variables is greater than the number of objects, as well as for the cross-correlation of the initial data. Moreover, the data series can be linear combinations of each other (Ränner et al., 1994).

## Results and discussion

A 2B-PLS model was built, the blocks of which included the following variables, which are series of instrumental data (13 variables, block No. 1) and series of features (26 variables, block No. 2) (see the Table). Accordingly, 13 latent structures were obtained.

**Tab. 1.**
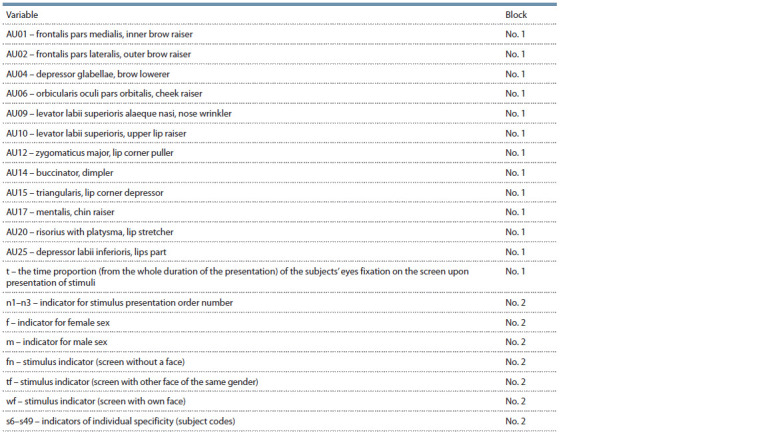
Blocks of variables for the 2B-PLS model Notе. AU classification is according to Facial Action Coding System (FACS).

**Fig. 2.**
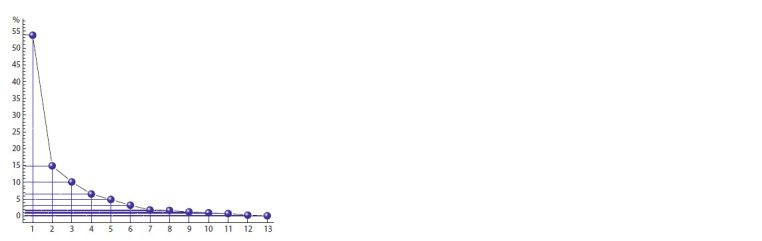
Scree plot for latent structures of the constructed 2B-PLS model. X-axis is the numbers of latent structures; Y-axis is the share of the observed total variance described by them.

As follows from the “scree” plot for latent structures of the constructed 2B-PLS model (Fig. 2), the first inflection of the graph falls on structure No. 2. Thus, structure No. 1 (before the first inflection) will reflect the general features of implicit cognitive processes (as it is confirmed by the proportion of observed total variance caused by it).

The second inflection of the graph falls on structure No. 4. Thus, for structures No. 2 and 3, the particular specificity of implicit cognitive processes will be defining. In the subsequent structures, the noise component grows simultaneously with a decrease in the share of the described total variance, however, we will also consider structure No. 4 – it causes more than 5 % of the total variance.

Later, the analysis of the results of psychological questionnaires, together with the results of clustering for the structures we obtained, can be used for the purposes of psychophysiological profiling of the subjects. Hence the conclusion that for subsequent profiling in the EEG experiment, it is necessary to assess the influence of individual differences of the subjects in their implicit cognitive processes when being presented with their own or someone else’s face

The first four latent structures describe 85.4 % of the total variance and the defining features are gender, stimulus type, and trial order.

**Fig. 3.**
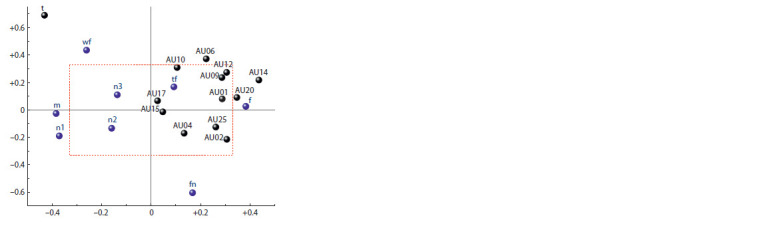
Loads (correlation coefficients) of variables for latent structure No. 1 (X-axis; 53.8 % of total variance) and structure No. 2 (Y-axis; 14.9 % of total variance) in 2B-PLS model. Here and in Fig. 4: black color – instrumental variables, blue color – feature variables (see the Table); inside the rectangle (red dotted line) the significance of the values of the correlation coefficients p > 0.05; markings of individual specificity are omitted to improve the readability of the graph.

According to Fig. 3, the first structure describes 53.8 % of the total variance and is determined by the proportion of the time the subject’s gaze is fixed on the screen, the activity of the buccinator and risorius muscles, gender characteristics, and the perception of all first trials. Hence, the perception of all the first samples is accompanied by an increase in activity of the buccinator and risorius muscles and a decrease in the proportion of the time of the gaze fixation on the screen in girls, and in boys – by a decrease in activity of said muscles and an increase in the proportion of the time of screen-fixed gaze.

The second structure describes 14.9 % of the total variance and is determined by the proportion of time the subject’s gaze is fixed on the screen, the activity of the cheek raiser muscle, and signs of the type of stimuli (see Fig. 3). Hence, the perception by all subjects of their own face on the screen is accompanied by an increase in activity of the cheek raiser and an increase in the proportion of time the gaze is fixed on the screen, while the perception of an empty screen – by a decrease in activity of the said muscle and a decrease in the proportion of time the gaze is fixed on the screen.

It can be noted that in the space of the first two latent structures, the perception of other face in all subjects is accompanied by an increase in the activity of the upper lip and chin raiser and the lip corner depressor.

**Fig. 4.**
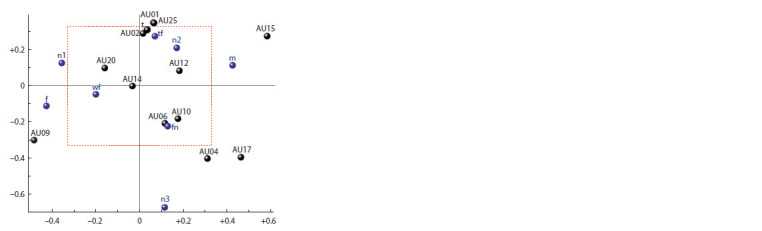
Load (correlation coefficients) of variables for latent structure No. 3 (X-axis; 10.1 % of total variance) and structure No. 4 (Y-axis; 6.6 % of total variance) in 2B-PLS model.

According to Fig. 4, the third structure describes 10.1 % of the total variance and is determined by the activity of the nose wrinkler, chin raiser, gender and first trial features. Hence it follows that the perception of all the first samples is accompanied by an increase in activity of nose wrinkle and a decrease in activity of chin raiser in girls, and in boys – by a decrease in activity of nose wrinkle and an increase in activity of chin raiser

The fourth structure describes 6.6 % of the total variance and is determined by the sign of the last trials, the activity of the inner brow raiser, the depressor glabellae muscles, the chin raiser, and parted lips (see Fig. 4). What can be inferred from this is that the reaction to all third trials in all subjects is accompanied by an increase in the activity of the chin raiser and the depressor glabellae muscles, a decrease in activity of the inner eyebrow raiser and the degree of relaxation of the chin muscle and the circular muscle of the mouth, and parted lips.

It can be noted that in the space of latent structures No. 3 and 4, the perception of other face in all subjects is accompanied by an increase in the proportion of the time of fixing the gaze on the screen and an increase in the activity of the inner and outer brow raiser, relaxation of the chin muscle and the circular muscle of the mouth, and parted lips.

Thus, in an EEG/ECG experiment, it is recommended for joint processing to include (apart from the influence of individual differences in implicit cognitive processes) the following covariate variables: gender, order of trials, presence of one’s own face on the screen/blank screen.

## Conclusions

Electroencephalogram is one of the most common methods for non-invasive study of the functional state of the human brain in healthy and clinical conditions. When analyzing the relationship between the EEG parameters and the behavioral activity of the subject, the motor (much less often verbal) responses of the subjects are usually chosen as behavioral metrics. This choice is primarily due to the fact that such responses are easy to mark in EEG recordings. We hypothesized that changes in the state of the facial muscles could serve as a behavioral phenotypic feature associated simultaneously with the personality characteristics of the survey participant, including their predisposition to mental disorders, and with endophenotypic parameters of brain rhythms.

In the present article, we propose a methodological idea for recording and processing facial video together with EEG recording. A pilot study was conducted aiming to find statistically significant covariates for facial expression to take into account in the analysis of EEG in the resting-state paradigm of functional rest and also when the subjects are being demonstrated a video recording of their own or someone else’s face. This approach is based on the face muscles dynamics analysis of the subject on video, which is recorded simultaneously with the registration of EEG and ECG.

It was shown that the dynamics of facial muscle activity reflect controlled conditions that are not usually used in the analysis of EEG correlates of cognitive processes, but which, as follows from the results, may accompany certain implicit cognitive processes. Taking into account such covariates as the subject’s gender, screen status (blank, own/other face) and sample number will increase the reliability of the assessment of the cognitive state of the subjects and provide additional information for interpreting the EEG/ECG results. The clustering of subjects by the factors of individual specificity of implicit cognitive processes will form a basis for effective profiling.

In the present study, we did not analyze EEG/ECG and psychometric data, as this is a pilot study with limited objectives. In the future, it is planned to increase the size of the experimental sample and conduct a more detailed comparison of the results of the analysis of the activity of the facial muscles with the results of other neurocognitive methods. For these promising tasks, we have worked out data obtaining methodology for profiling subjects according to the latent structures described by the authors, which allows to use the results of the generated model as additional variables for second-level summary models (including EEG, ECG data, etc.).

## Conflict of interest

The authors declare no conflict of interest.
